# Matrix metalloproteinase 1 and circulating tumor cells in early breast cancer

**DOI:** 10.1186/1471-2407-14-472

**Published:** 2014-06-28

**Authors:** Zuzana Cierna, Michal Mego, Pavol Janega, Marian Karaba, Gabriel Minarik, Juraj Benca, Tatiana Sedlácková, Silvia Cingelova, Paulina Gronesova, Denisa Manasova, Daniel Pindak, Jozef Sufliarsky, Ludovit Danihel, James M Reuben, Jozef Mardiak

**Affiliations:** 1 Department of Pathology, Comenius University, Bratislava, Slovakia; 2 Translational Research Unit, Comenius University, Bratislava, Slovakia; 3 2nd Department of Medical Oncology, Comenius University, Faculty of Medicine, National Cancer Institute, Klenova 1, 833 10 Bratislava, Slovak Republic; 4 Institute of Molecular Biomedicine, Faculty of Medicine, Comenius University, Bratislava, Slovakia; 5 National Cancer Institute, Bratislava, Slovakia; 6 Institute of Normal and Pathological Physiology, Bratislava, Slovakia; 7 Cancer Research Institute, Slovak Academy of Sciences, Bratislava, Slovakia; 8 Slovak Medical University, Bratislava, Slovakia; 9 Department of Hematopathology, The University of Texas MD Anderson Cancer Center, Houston, Texas, USA

**Keywords:** Circulating tumor cells, Matrix-metaloproteinase 1, Primary breast cancer

## Abstract

**Background:**

Matrix metalloproteinases (MMPs) are involved in cancer invasion and metastasis. Circulating tumor cells (CTCs) play role in tumor dissemination and are an independent survival predictor in breast cancer (BC) patients. The aim of this study was to assess correlation between CTCs and tumor MMP1 in BC.

**Methods:**

Study included 149 primary BC patients treated by surgery from March 2012 to March 2013. Peripheral blood mononuclear cells (PBMC) were depleted of hematopoietic cells using RossetteSep^TM^ selection kit. RNA extracted from CD45-depleted PBMC was interrogated for expression of EMT (TWIST1, SNAIL1, SLUG, ZEB1) and epithelial (CK19) gene transcripts by qRT-PCR. Patient samples with higher epithelial and/or mesenchymal gene transcripts than those of healthy donors (n = 60) were considered as CTC positive. Expression of MMP1 in surgical specimens was evaluated by immunohistochemistry.

**Results:**

CTCs were detected in 24.2% patients. CTCs exhibiting only epithelial markers were present in 8.7% patients, whereas CTCs with epithelial-mesenchymal transition (EMT) markers (CTC_EMT) were observed in 13.4% of patients and CTCs co-expressing both markers were detected in 2.0% patients. Patients with CTC_EMT in peripheral blood had significantly increased expression of MMP1 in tumor cells (p = 0.02) and tumor associated stroma (p = 0.05) than those of patients without CTC_EMT. In multivariate analysis, CTC_EMT and tumor grade were independently associated with MMP1 expression in cancer cells, while CTC_EMT and Ki67 were independently associated with MMP1 expression in cancer associated stroma.

**Conclusion:**

Our data suggest link between MMP1 and CTCs with EMT phenotype and support role of MMPs and EMT in tumor dissemination.

## Background

Circulating tumor cells (CTCs) play an important role in the metastatic cascade, cancer dissemination and progression. Prognostic value of CTCs was showed by numerous trials for metastatic as well as primary breast cancers [1-4]. CTCs constitute a heterogeneous population of cells with different phenotypes, clinical and biological value [5].

Invasion is one of the first critical steps in the metastatic cascade that requires changes in cell-to-cell adhesion and cell adhesion to the extracellular matrix (ECM). Invasion is often accompanied by downregulation of epithelial molecules on cell surface including E-cadherin, and upregulation of mesenchymal molecules like N-cadherin, which is closely associated with mesenchymal cells [6]. Invasion is further facilitated by proteolytic degradation of the ECM, which enables cancer cells to penetrate tissue boundaries. Degradation of the ECM is predominately mediated by matrix metalloproteinases (MMPs) and the urokinase plasminogen activator (uPa) system [7].

Matrix metaloproteinase 1 (MMP1) is a zinc-dependent endopeptidase with collagen-cleaving activity. MMP1 cleave extracellular matrix components and thus play an important role in tumor invasion [8]. MMP1 is produced by tumor cells as well as by tumor associated stroma. High MMP1 expression in tumor is associated with tumor evolution, poor prognosis and shortened survival in different types of tumors including breast cancer [9,10]. Many studies suggest that overexpression of MMPs is one of the key events leading to the breast cancer dissemination. Recently, it was shown, that MMPs induce epithelial to mesenchymal transition and thus increase the invasive potential of tumor cells [11,12].

In this translational study, we hypothesized, that MMP1 is involved in CTC release and CTCs are detected more often in breast cancer patients with high MMP1 expression in primary tumor or tumor associated stroma. Therefore, we examined expression of MMP1 on breast tumor tissue as well as tumor associated stroma and correlated them with CTCs in peripheral blood. We also correlated MMP1 expression with other patients’ tumor characteristics.

## Methods

### Study patients

As a part of ongoing translational study (Protocol TRU-SK 002; Chair: M. Mego), treatment naive patients with stages I–III primary breast cancer (PBC) who were undergoing definitive surgery were included. From each patient we obtained peripheral blood for CTCs detection on the day of surgery and corresponding paraffin-embedded tumor tissue. Each patient was given a complete diagnostic evaluation to exclude the presence of distant metastasis. Patients with concurrent malignancy other than non-melanoma skin cancer in the previous 5 years were excluded as well. In all patients, data regarding age, tumor stage, histology, regional lymph node involvement, hormone receptor status, and HER2 status were also recorded. All patients agreed to participate in the study and signed informed consent according to the IRB-approved protocol.

The study was approved by the Institutional Review Board (IRB) of the National Cancer Institute of Slovakia and patients were enrolled between March 2012 and March 2013. Healthy donors (N = 60) were age-matched women without breast cancer who were recruited and consented according to the IRB-approved protocol. Healthy donors were recruited from staff working in cooperating institutions except women from departments directly involved in the study.

### Detection of CTC in peripheral blood

CTC were detected in peripheral blood by quantitative real-time polymerase chain reaction (qRT-PCR) based assay utilizing CD45 positive (CD45+) cells depletion for CTCs enrichment, as described previously [13].

#### RNA extraction

Peripheral blood was subjected to CD45 depletion using RossetteSep™ kit (StemCell technologies) according to the manufacturer’s instructions. CD45-depleted cells were mixed with TRIzolVR LS Reagent (Invitrogen Corporation, Carlsbad, CA) and stored at -80°C until it was necessary to extract RNA according to the manufacturer’s instructions. All RNA preparation and handling steps took place in a laminar flow hood, under RNase-free conditions. RNA concentration was determined by absorbance readings at 260 nm.

#### Identification of gene transcripts in CD45-enriched subsets

Isolated RNA was subjected to quantitative RT-PCR (qRT-PCR) to detect EMT-inducing transcription factors (EMT-TF) gene transcripts (TWIST, SNAIL1, SLUG and ZEB1) and epithelial antigen (CK19). In brief, 2.5 μL of cDNA were placed in 25 μL of reaction volume containing 12.5 μL of QuantiFast Probe RT-PCR Kit Master Mix, 0.25 μL QuantiFast RT Mix, 8.5 μL water and 1.25 μL of primers. The following TaqMan assays were purchased from LifeTechnologies (USA): Twist1: Hs00361186_m1; Snail1: Hs00195591_m1; Slug: Hs00161904_m1; Zeb1: Hs01566408_m1; Gapdh Hs99999905_m1; Ck19 Hs00761767_s1. Amplicons or probes spanned intron–exon boundaries, with the exception of CK19. Amplification was performed on an Eppendorf Realplex Real-Time PCR system (Eppendorf, Germany) using the cycling program: 95°C for 10 min; 40 cycles of 95°C for 15 sec and 60°C for 60 sec. All samples were analyzed in triplicate. Calibrator samples were run with every plate to ensure consistency of the PCR. For all fluorescence-based RT-PCR, fluorescence was detected between 0 and 40 cycles for the control and marker genes in single-plex reactions, which allowed for the deduction of the cycles at threshold (Ct) value for each product. Expression of the genes of interest was calibrated against expression of the housekeeping gene, GAPDH. Target cDNA was quantified using the delta-Ct method with the formula: 1 = 2 Ct(target-GAPDH).

#### CTC definition

Patient samples with higher Ck19 gene transcripts than those of healthy donors were considered as epithelial CTCs positive (CTC_EP), while patient samples with higher EMT-TF (Twist1, Snail1, Slug and Zeb1) gene transcripts than those of healthy donors were considered as CTC_EMT positive.

The highest expression levels of the CK19 and EMT-inducing TF gene transcripts relative to that of Gapdh were 3.4 × 10 ^-3^, 2.0 × 10^-4^, 1 × 10^-2^ and 2.2 × 10^-2^ for Ck19, Twist1, Snail1 and Zeb1, while Slug transcripts were not detected in any of the samples from healthy donor. These values were used as “cutoff” to determine CTCs positivity.

### Tumor pathology

Pathology review was conducted at the Department of Pathology, Faculty of Medicine, Comenius University, by pathologists (ZC, LD and PJ) associated with the study.

### Diagnosis and tumor samples

The study included tumor specimens from 149 patients. All specimens were classified according to the WHO Classification of 2004. The block containing the most representative part of the haematoxylin and eosin (H&E) stained tumor was identified by microscopy and subsequently used for IHC analysis.

### Tissue microarray construction

According to tumor histology, one or two representative tumor areas were identified on H&E stained sections. Sections were matched to their corresponding wax blocks (the donor blocks), and 3-mm diameter cores of the tumor were removed from donor blocks with the multipurpose sampling tool Harris Uni-Core (Sigma-Aldrich, Steinheim, Germany) and inserted into the recipient master block. The recipient block was cut into 5-μm sections, and the sections were transferred to coated slides.

### Immunohistochemical (IHC) staining

Slides were deparaffinised and rehydrated in phosphate buffered saline solution (10 mM, pH 7.2). The tissue epitopes were demasked using the automated water bath heating process in Dako PT Link (Dako, Glostrup, Denmark); the slides were incubated in pH 6.0 citrate retrieval buffer at 98°C for 20 minutes. The slides were subsequently incubated overnight at room temperature with the primary rabbit polyclonal antibody against MMP1 (LSBio, MMP-1, LS-B1229) diluted 1:40 in Dako REAL antibody diluent (Dako, Glostrup, Denmark) and immunostained using anti-mouse/anti-rabbit immuno-peroxidase polymer (EnVision FLEX/HRP, Dako, Glostrup, Denmark) for 30 minutes at room temperature, according to the manufacturer’s instructions. For visualisation, the slides were reacted with diaminobenzidine substrate-chromogen solution (DAB, Dako, Glostrup, Denmark) for 5 minutes. Finally, the slides were counterstained with haematoxylin. As negative control, breast tissue was subjected to the same staining procedure without reaction with the primary antibody.

### Immunohistochemical stain scoring

Tumor cores were independently assessed by pathologists (ZC, LD and PJ) who were blinded to clinico-pathological data. In cases of disagreement, the result was reached by consensus. The result of the immunohistochemical analyses was expressed by a weighted histoscore, evaluating both the percentage of positive cells (PP) and the staining intensity (SI) of the nuclei or cytoplasm. Briefly, the proportion of cells with nuclear staining was multiplied by the intensity of staining to provide a histoscore ranging from 0–300. The histoscore was calculated as follows: Score = (0 × percentage not stained) + (1 × percentage weakly stained) + (2 × percentage moderately stained) + (3 × percentage strongly stained) [14].

### Statistical analysis

Patient characteristics were tabulated. The patients’ characteristics were summarized using the median (range) for continuous variables and frequency (percentage) for categorical variables. Normality of distribution was tested by the Kolmogorov-Smirnoff test. If normally distributed, sample means were tested by Student *t*-test or analysis of variance (ANOVA) with Bonferroni’s or Tamhane’s corrections, depending on homogeneity of variance. Nonparametric Mann–Whitney *U* or Kruskal-Wallis H test were used for non-normally distributed data. A multivariate linear regression model was built using a step-wise regression technique, regression coefficients remained in the model at significance level of 0,05. MMP1 expression levels were analyzed as continuous variables. Because the MMP expression levels were highly skewed, Box-Cox transformations [15] were used for the determination of p-values, with optimum λ = 0,19 for MMP1 in tumor stroma and optimum λ = 0,16 for MMP1 expression in tumor cells. All p values presented are two-sided, and associations were considered significant if the p value is less or equal to 0.05. Statistical analyses were performed using NCSS 2007 software (Hintze J, 2007, Kaysville, Utah, USA).

## Results

The study population consisted of 149 primary breast cancer patients with median age of 60 years (range: 31–83 years). Patients’ characteristics are shown in Table 1. The majority of patients had hormone receptor positive (87.2%), node negative (61.1%) tumors; 22 (14.8%) patients had HER-2/neu amplified tumors.

**Table 1:** Patients characteristics

**Variable**	**N**	**%**
**All**	149	100.0
**T-stage**		
**1**	105	70.5
**> 1**	44	29.5
**N-stage**		
**0**	91	61.1
**> 1**	58	38.9
**Grade**		
**1 and 2**	95	63.8
**3**	54	36.2
**Histology**		
**IDC**	128	85.9
**Other**	21	14.1
**Hormone receptor status**		
**Negative**	19	12.8
**Positive**	130	87.2
**HER2 status**		
**Negative**	127	85.2
**Amplified**	22	14.8
**Ki 67 (cut-off 20%)**		
**Low**	92	61.7
**High**	57	38.3
**Lymphovascular invasion**		
**Present**	35	23.5
**Absent**	114	76.5
**Baseline CTC**		
**CTC Epithelial**		
**Negative**	133	89.3
**Positive**	16	10.7
**CTC EMT**		
**Negative**	126	84.6
**Positive**	23	15.4
**CTC Any**		
**Negative**	113	75.8
**Positive**	36	24.2

### CTC detection

To determine overexpression of the EMT-TF gene transcripts and Ck19 in PBC patients, we compared the expression levels in patient samples with those of HDs. Relative to the highest levels of Snail and Zeb1 transcripts detected in HD samples, none of the patient samples overexpressed these gene transcripts. Among the patient samples, Twist1, Slug, and Ck19 transcripts were overexpressed in 3 (2.0%), 20 (13.6%) and 16 (10.7%) samples, respectively. Totally, CTCs were detected in 36 (24.2%) of patients. CTCs with only epithelial markers were present in peripheral blood of 13 (8.7%) patients; CTCs with EMT phenotype were present in 20 (13.4%) of patients, while in 3 (2.0%) of patients CTCs exhibited both epithelial and mesenchymal markers. In one patient sample, there was overlap in overexpression of EMT-TF gene transcripts (Slug and Twist1).

### Correlation between MMP1 expression and CTC and patients’/tumor characteristics

**Figure 1:**
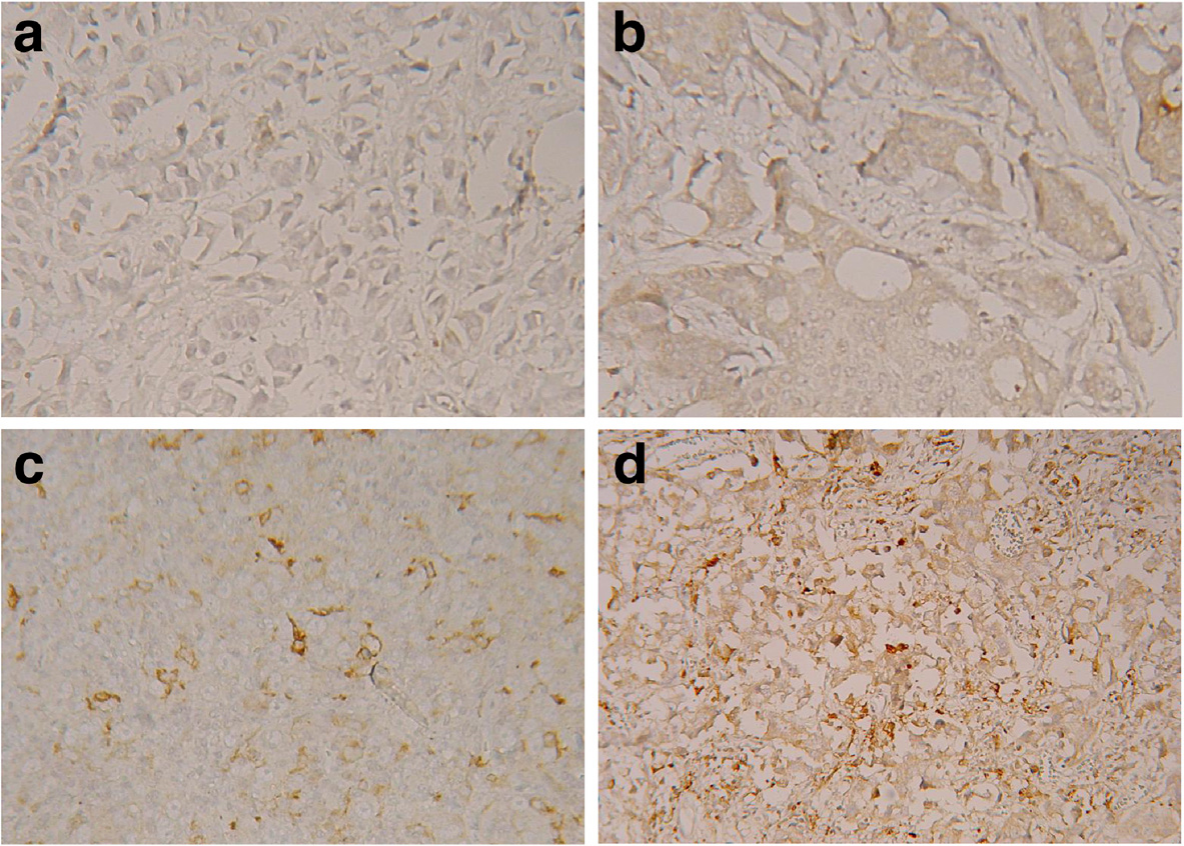
**MMP1 expression in primary breast tumors.** Immunohistochemical reaction with anti-MMP1 monoclonal antibody. Original magnification × 400 visualisation with 3,3′-diaminobenzidine. **a)** staining intensity 0, **b)** staining intensity 1+, **c)** staining intensity 2+, **d)** staining intensity 3 + .

MMP1 expression at least 1+ and higher was detected in 104 (69.8%) of samples in breast tumor cells and in 120 (80.5%) samples of tumor associated stroma (p = 0.04) (Figure 1). Mean ± SEM (standard error of mean) for MMP1 expression histoscore in tumor associated stroma was significantly higher compared to breast tumor cells (32.8 ± 3.4 vs. 49.1 ± 5.1, p = 0.05). Expression of MMP1 in relation to CTCs and various clinicopathological characteristics is shown in Table 2.

**Table 2:** MMP1 expression in tumor cells and tumor stroma

**Variable**		**MMP1 expression histoscore in tumor cells**				**MMP1 expression histoscore in tumor associated stroma**			
	**N**	**Mean**	**SEM**	**Median**	**P-value**	**Mean**	**SEM**	**Median**	**P-value**
**All**	149	32.8	3.4	15	NA	49.1	5.1	20	NA
**T-stage**									
**1**	105	30.6	4.0	14	0.28	49.7	6.1	20	0.76
**> 1**	44	44.0	38.2	17		43.0	47.6	20	
**N-stage**									
**0**	91	32.1	4.4	14	1.00	44.7	6.5	20	0.29
**> 1**	58	34.0	5.5	18		55.9	8.1	20	
**Grade**									
**1 and 2**	95	27.0	4.2	10	0.007	37.9	6.2	6	0.0006
**3**	54	43.2	5.6	30		69.2	8.3	50	
**Histology**									
**IDC**	128	34.3	3.7	20	0.15	53.8	5.4	20	0.0022
**Other**	21	23.6	9.0	4		20.8	13.3	1	
**Hormone receptor status**									
**Negative**	19	42.0	9.5	20	0.26	70.7	14.5	50	0.18
**Positive**	130	31.5	3.6	13		46.1	5.4	20	
**HER2 status**									
**Negative**	127	30.4	3.7	10	0.18	45.0	5.5	20	0.29
**Amplified**	22	47.1	8.8	25		72.5	13.1	40	
**Ki 67 (cut-off 20%)**									
**Low**	92	28.6	4.3	10	0.04	34.7	6.2	10	0.0006
**High**	56	39.7	5.5	25		72.8	7.9	50	
**Lymphovascular invasion**									
**Present**	35	41.0	7.0	20	0.10	48.2	10.5	20	0.83
**Absent**	114	30.3	3.9	11		49.4	5.8	20	
**Baseline CTC**									
**CTC Epithelial**									
**Negative**	133	32.9	3.6	16	0.35	48.6	5.4	20	0.50
**Positive**	16	32.1	10.4	3		53.0	15.5	11	
**CTC EMT**									
**Negative**	126	30.2	3.7	10	0.02	45.7	5.5	20	0.05
**Positive**	23	47.0	8.6	40		68.5	13.1	50	
**CTC Any**									
**Negative**	113	31.3	3.9	10	0.47	47.9	5.8	20	0.75
**Positive**	36	37.5	6.9	20		53.0	10.5	20	

We observed an association between CTC_EMT and expression of MMP1 in breast cancer cells as well as in cancer associated stroma (Table 2), and found no correlation between CTC_EP and MMP1 expression. MMP1 expression in breast cancer cells was associated with high tumor grade, and increased proliferation (high Ki67), while MMP1 expression in tumor associated stroma, besides high tumor grade and increased proliferation, correlated with invasive ductal histology (Table 2). There was no association between expression of MMP1 and ER/PR status, HER2/neu amplification or axillary lymph node status (Table 2).

In multivariate analysis CTC_EMT (p = 0.03) and tumor grade (p = 0.01) were independently associated with MMP1 expression in breast tumor cells, while Ki-67 (p = 0.0001) and CTC_EMT (p = 0.04) were independently associated with MMP1 expression in tumor associated stroma (Tables 3 and 4).

**Table 3:** Multivariate logistic regression model for expression of MMP1 in tumor cells

**Parameter**	**T Statistic**	**P-value**
**Tumor grade (1 and 2 vs. 3)**	2.59	0.0106
**CTC_EMT present vs. absent**	2.22	0.0277

**Table 4:** Multivariate logistic regression model for expression of MMP1 in tumor associated stroma

**Parameter**	**T Statistic**	**P-value**
**Ki 67 (high vs. low)**	3.94	0.0001
**CTC_EMT present vs. absent**	2.03	0.0444

## Discussion

In this translational study, we showed association between CTC with EMT phenotype (CTC_EMT) and MMP1 expression in primary breast cancer. This association was observed for MMP1 expression in cancer cells as well as in cancer associated stroma. MMP1 expression was increased in primary tumors with poor prognostic features such as high grade tumors with increased proliferation (Ki 67 > 20%) which is consistent with previous observations [9]. We also found association between ductal carcinoma histology and MMP1 expression as described previously [16]; however, in our study, this association was statistically significant only for tumor associated stroma.

Breast cancer represents highly complex tissue composed of cancer cells and stromal cell compartments containing different types of mesenchymal cells. MMPs, in breast cancer, are produced by cancer cells as well as cancer associated stroma and are involved in cancer progression through multiple mechanisms. Degradation of ECM by MMPs facilitates movement of cancer cells through ECM. MMPs also disrupt cell-cell and cell-ECM adhesions that result in the release of individual tumor cells from epithelial sheets, and initiating signaling pathways that lead to widespread changes in gene expression patterns that are responsible for increased migration and invasion of breast cancer cells [7,11,17]. MMPs could also impact cancer cell behaviour due to their ability to cleave growths factors, cell surface receptors, cell adhesion molecules and chemokines [7,16]. Therefore, MMPs are promising therapeutic targets in breast cancer and several trials evaluating drugs that interfere with MMPs function are ongoing [reviewed by 11].

EMT is believed to play an important role in intravasation and the release of CTCs, and the expression of EMT-inducing TF gene transcripts in breast cancer has been associated with poor prognosis [18]. EMT has been previously linked with cancer stem cell properties [19] which have been associated with increased therapeutic resistance [20-22]. MMPs have been associated with EMT in cancer progression by several mechanisms [11]. Elevated levels of MMPs in the tumor microenviroment can directly induce EMT in epithelial cells. Cancer cells that undergo EMT could produce more MMPs further supporting cell invasion and metastasis and finally, EMT can generate activated stromal like cells that drive cancer progression via further MMPs production [11]. The most important of these is MMPs mediated activation of EMT that was seen in variety of epithelial tumors and has been best characterized in mammary epithelial cells [11]. Our observation thus further support association between MMPs and EMT in breast cancer.

EMT of tumor cells could produce stromal-like cells that could further facilitate tumor progression through the production of MMPs. Myofibroblasts are key components of cancer associated stroma in breast cancer and these cells have important tumor promoting activity. Myofibroblasts can be derived through activation of stromal fibroblast or circulating fibrocytes; however, recent studies in mouse models have shown, that myofibroblasts can be derived from epithelial cells by EMT, as well [23-26]. In our study, we observed an association between CTC_EMT and MMP1 expression in both cancer cells as well as in cancer associated stroma. Expression of MMP1 in the stromal compartment of breast carcinomas possibly represents two populations of cells: EMT transformed neoplastic cells and stromal fibroblastic cells that undergo activation of EMT induced TFs due to growth factors produced by the tumor [27]. However, we don’t know exactly, if an observed association between CTC_EMT and MMP1 in tumor stroma, was due to expression of MMP1 in stromal fibroblasts, or stromal like cancer cells that underwent EMT.

The flow of cancer cells may not be unidirectional. Experimental data suggest, that CTCs can be released from metastatic tumors and then rejoin the tumor of origin, a process termed ‘self seeding’ [28,29]. It seems, that self-seeding is a major driver of tumor progression in solid tumors and that CTCs are mediators of tumor self-seeding [28]. CTCs are an independent prognostic factor in breast cancer and probably represent surrogate marker for tumor self-seeding ability. In seminal work, Kim et al. observed that MMP1 is a mediator of CTCs infiltration into mammary tumors [28] and are involved in tumor self-seeding in an animal model. Our translational data are consistent with these observations and further support role of MMP1 in tumor self-seeding.

The EMT is a dynamic and progressive process, and it can also be reversible. Therefore, it usually produces cells with a spectrum of intermediate phenotypic states. Cells can advance to differing extents through an EMT program, progressively acquiring mesenchymal features as they shed epithelial ones [5]. Cells that have entered an EMT program rarely shed all of their pre-existing epithelial features. In the context of carcinoma pathogenesis, neoplastic cells may reside in a state in which they coexpress newly acquired mesenchymal markers together with retained epithelial ones [13,30,31]. For these reasons our distinction of two CTCs subpopulation (CTC-EP, CTC-EMT) may represent some study limitations.

## Conclusion

In conclusion, in this prospective translational study, we for the first time showed association between CTC_EMT and expression MMP1 in primary tumor tissue. We suppose that the therapeutical targeting of MMP1 could lead to decreased MMP1-induced EMT and subsequently, to decrease of CTC_EMT, with implications for tumor dissemination and treatment resistance. Future studies will be needed to identify expression of other proteins in tumor tissue associated with presence of CTCs in the peripheral blood. These proteins could represent surrogate markers for biologically more aggressive disease and could represent potentially new therapeutic targets to inhibit metastatic process.

## Ethics approval

The Institutional Review Board of the National Cancer Institute, Bratislava, Slovakia.

## Abbreviations

ANOVA: Analysis of variance; CD45+: CD45 positive; CTCs: Circulating tumor cells; ECM: Extracellular matrix; EMT: Epithelial-to-mesenchymal transition; EMT-TF: EMT-inducing transcription factors; ER: Estrogen receptor; HD: Healthy donors; H&E: Hematoxyllin and eosin; IRB: Institutional Review Board; IRS: ImmunoReactive Score; MBC: Metastatic breast cancer; MMPs: Matrix metalloproteinases; PB: Peripheral blood; PBMC: Peripheral blood mononuclear cells; PR: Progesterone receptor; qRT-PCR: Quantitative real time polymerase chain reaction; SEM: Standard error of the mean; TF: Tissue factor.

## Competing interest

On behalf of all the authors I declare that there are no competing financial interests in relation to the work described in the manuscript.

## Authors’ contribution

M-M, J-R, J-S and J-M participated in conception and design of this study. Tumor cores were independently assessed by pathologists (ZC, LD and PJ) who were blinded to clinicopathological data. S-C performed statistical analysis, G-M, T-S, D-M were involved in CTCs detection, M-K, J-B, D-P were involved in patients accrual and performed breast surgery. M-M and ZC drafted the article and all authors reviewed it critically for important intellectual content. All the authors participated in the aquisition, analysis and interpretation of data. All the authors gave their final approval of the version to be published.

## Authors’ information

Cierna Z and Mego M share first authorship.

## Pre-publication history

The pre-publication history for this paper can be accessed here:

http://www.biomedcentral.com/1471-2407/14/472/prepub
